# Analysis of the therapeutic efficacy of OLIF combined with posterior percutaneous pedicle screw fixation in the treatment of patients with primary lumbar spondylodiscitis

**DOI:** 10.3389/fsurg.2025.1486695

**Published:** 2025-02-25

**Authors:** Liang Deng, Yu Zhou, Moliang Xiong, Jihuan Zeng, Caiguang Luo, Jia Guo, Qiang Xiao

**Affiliations:** ^1^ Department of Orthopedics, Jiangxi Provincial People’s Hospital, The First Affiliated Hospital of Nanchang Medical College, Nanchang, China; ^2^ Department of Orthopedics, Anfu Prefecture Hospital of Traditional Chinese Medicine, Ji'an, Jiangxi, China

**Keywords:** primary lumbar spondylodiscitis, oblique lateral interbody fusion (OLIF), posterior percutaneous pedicle screw fixation, catheter irrigation and drainage, JOA and VAS scores

## Abstract

**Introduction:**

Primary lumbar spondylodiscitis is a serious condition with an increasing incidence rate.

**Methods:**

From January 2021 to June 2023, we adopted a single-stage oblique lateral approach for the debridement of lumbar infection foci, intervertebral bone grafting fusion, tube irrigation drainage, combined with posterior percutaneous pedicle screw fixation for the treatment of primary lumbar spondylodiscitis.

**Results:**

We found that this surgical technique significantly improved the patients' lower back pain symptoms. During postoperative follow-ups at 1, 3, 6, and 12 months, patients showed remarkable improvements in their JOA and VAS scores, as well as in ESR and CRP levels, compared to the preoperative period.

**Discussion:**

We believe that the single-stage oblique lateral approach for debridement of lumbar infection foci, intervertebral bone grafting fusion, tube irrigation drainage, and combined posterior percutaneous pedicle screw fixation is an ideal treatment method for primary lumbar spondylodiscitis. This technique offers thorough debridement of the infection focus, sufficient bone grafting, safe operation, and reliable fixation.

## 1 Background

Primary spondylodiscitis is a serious condition and also one of the main causes of lower back pain. It can lead to numerous complications, such as chronic pain, spinal instability, neurological deficits, and in severe cases, death (1).The incidence rates reported in the existing literature vary significantly (2). In recent years, there has been a clear upward trend in the incidence of primary spondylodiscitis, with risk factors including diabetes, long-term use of steroids, and others. Additionally, there is a certain correlation with the aging population and advances in diagnostic technology (3). Currently, there is no definitive standard treatment protocol for primary spondylodiscitis. Conservative treatment is preferred, but it often requires a long duration and has a high recurrence rate. The concept of early surgical intervention has been widely accepted. Early surgical treatment can more effectively clear the infection, decompress the spinal canal, stabilize the spine, and restore potential neurological deficits (1, 4).

Oblique Lumbar Interbody Fusion (OLIF) is a minimally invasive surgical technique that has been developed in recent years and is now widely used globally. It involves accessing the intervertebral disc through the space between the peritoneum and the psoas muscle. Compared to other spinal fusion procedures, OLIF has the advantages of shorter operation time, less blood loss, quicker recovery, and reduced hospital stay (5). January 2021 to June 2023, our department employed the OLIF approach for a one-stage oblique lateral route to clear infection foci, perform intervertebral bone grafting fusion, and place a tube for irrigation and drainage, in combination with percutaneous pedicle screw fixation from the posterior approach to treat 26 patients with primary lumbar intervertebral space infection. The clinical outcomes were satisfactory, and the report follows.

## 2 Materials and methods

### 2.1 Case selection criteria

#### 2.1.1 Inclusion criteria

(1) Symptoms, signs, laboratory, and imaging findings consistent with primary lumbar spondylodiscitis, with etiological and pathological confirmation of infectious lesions; (2) Acute, subacute, and chronic lumbar spondylodiscitis that show no improvement or poor response after 2 weeks of conservative treatment; (3) Infection localized to a single segment of lumbar spondylodiscitis and adjacent endplates, with the integrity of the posterior spinal structures; (4) Spinal instability, abscess formation.

#### 2.1.2 Exclusion criteria

(1) Multi-segment lumbar spondylodiscitis, with large paravertebral abscesses that are difficult to completely clear through a posterior approach; (2) Severe spinal nerve compression requiring posterior spinal canal decompression; (3) Extensive peritoneal adhesion due to a history of retroperitoneal surgery, affecting the establishment of the surgical approach; (4) Elderly patients with multiple underlying diseases who are unable to tolerate surgery.

### 2.2 General information

**Figure 1:**
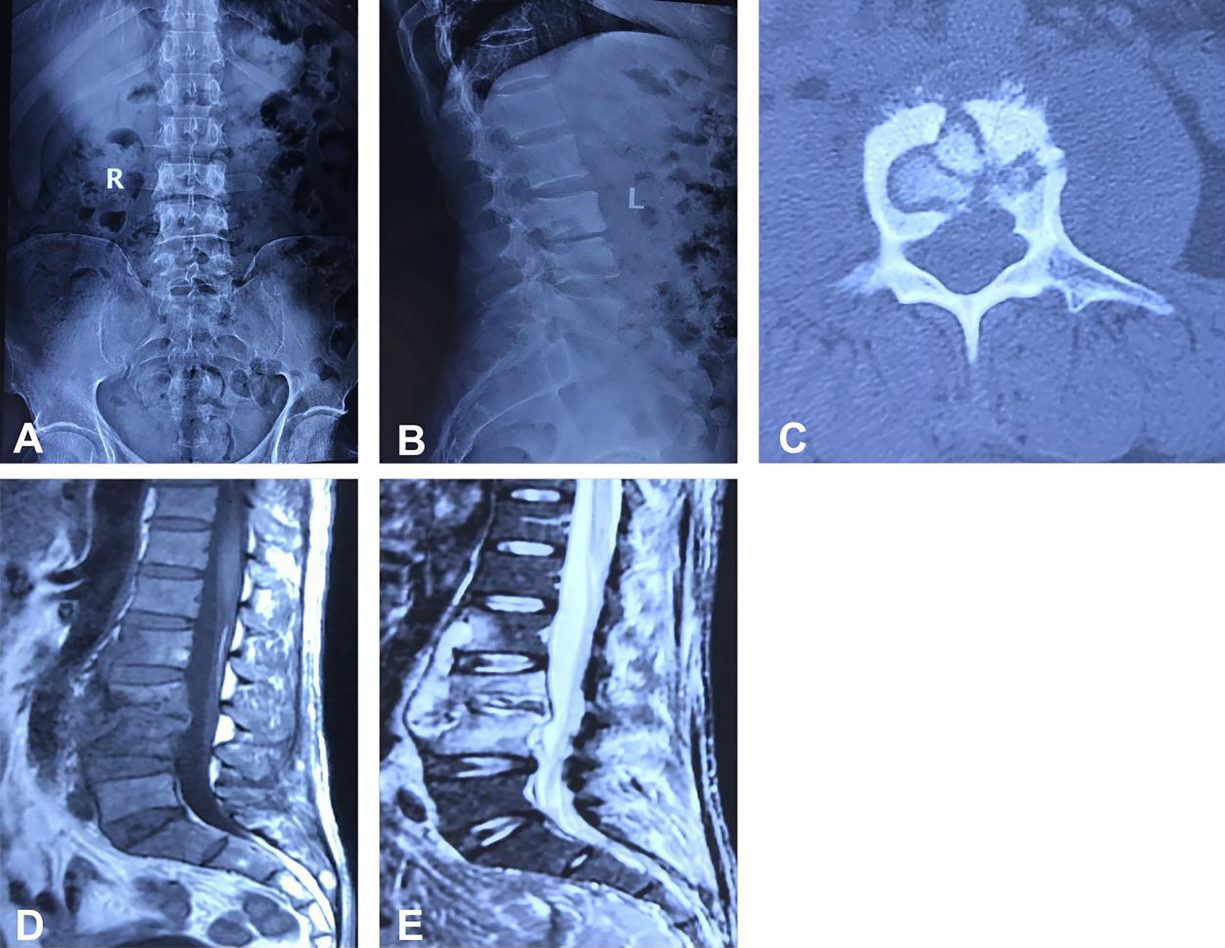
Depicts the case of a 71-year-old male patient with an L3/4 intervertebral space infection. Preoperative imaging, including anteroposterior and lateral x-rays, CT, and MRI scans, are presented in panels **(A–E)**. The images reveal an infection at the L3/4 intervertebral space, destruction of the bony structure of the L3/4 vertebrae, and the formation of a paravertebral abscess.

This study included a total of 26 patients, with 16 males and 10 females, ranging in age from 46 to 82 years (average age 65 ± 9.8 years). All patients had a single lumbar spondylodiscitis, with 6 cases at L2/3, 8 cases at L3/4 (Figure 1), and 12 cases at L4/5. Among these patients, 12 cases had concurrent diabetes, 13 cases had hypertension, and 4 cases had pulmonary tuberculosis. None of the patients had a history of lumbar spine surgery or trauma. The primary clinical manifestation was back pain accompanied by varying degrees of lower limb neurological dysfunction.

### 2.3 Surgical method

All surgeries were performed by the same team of doctors and were conducted under general anesthesia. The procedures involved a single-stage oblique lateral approach for the debridement of lumbar spine infections, intervertebral bone grafting fusion, placement of tube irrigation drainage, and combined posterior percutaneous pedicle screw internal fixation.

#### 2.3.1 Oblique lateral approach for the debridement of lumbar spine infections, intervertebral bone grafting fusion and placement of tube irrigation drainage

**Figure 2:**
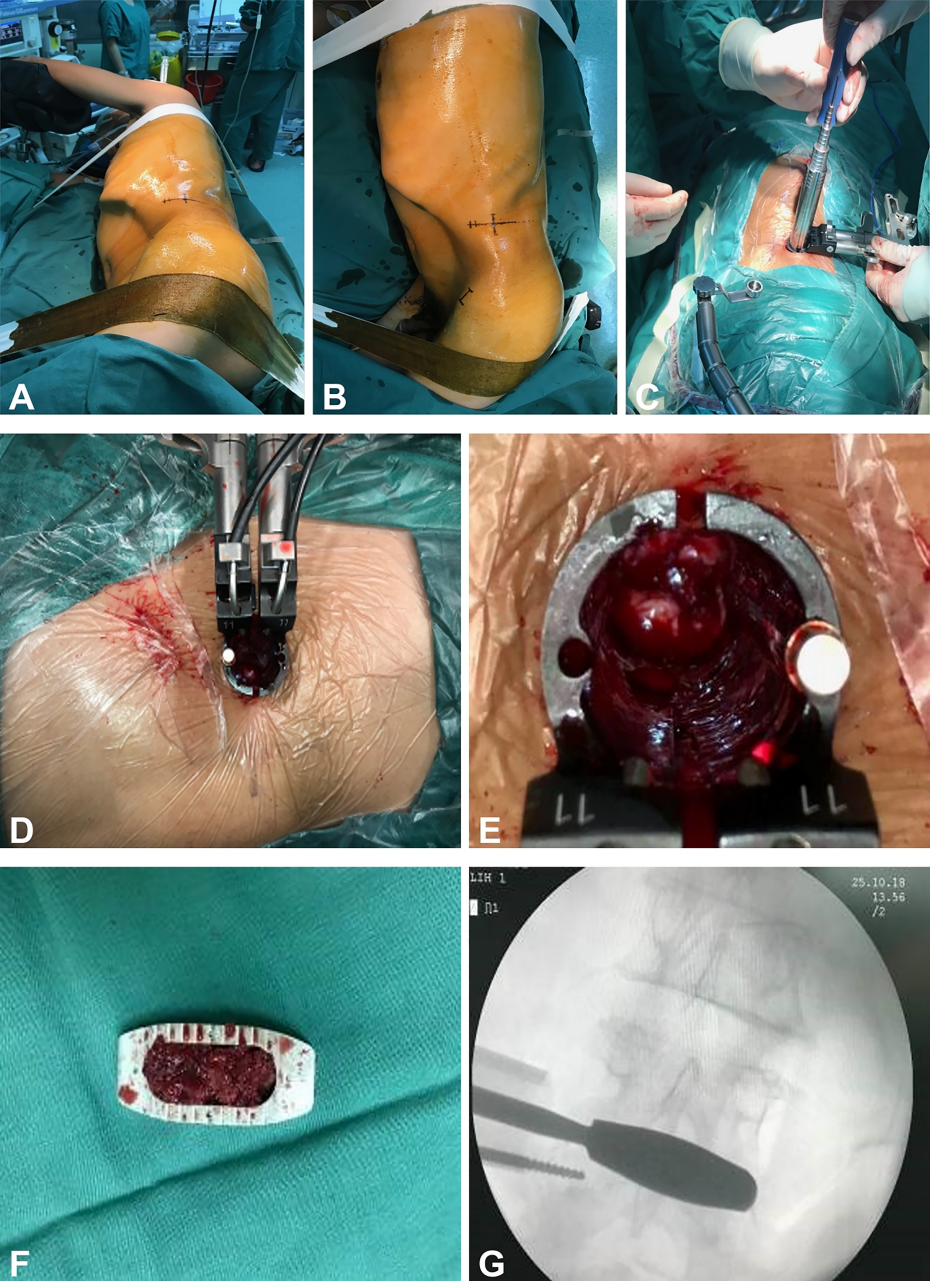
**(A–G)** Depicts the patient in a lateral decubitus position. After disinfection and draping, an incision is made according to the marked points to establish a working channel. Subsequently, the infected interspace is exposed, and following the thorough debridement of the infected tissue, an intervertebral fusion device is implanted.

After satisfactory anesthesia, the patient is positioned laterally with the side of the body perpendicular to the operating table and secured with 6–8 cm wide adhesive tape. The C-arm x-ray machine is used to determine the level of the diseased intervertebral space and the anterior and posterior edges of the vertebral body, and markings are made with a marker pen, followed by routine disinfection and draping. A skin incision about 4 cm long is made along the marked line, successively cutting through the skin, subcutaneous tissue, and the external oblique aponeurosis. Blunt dissection is used to open the internal oblique, transversus abdominis, and transversalis fascia, entering the retroperitoneal space. Fingers are used for blunt dissection in the retroperitoneum, pushing the retroperitoneal fat and abdominal organs towards the abdominal side, reaching the lateral edge and front of the vertebral body, and pushing back the psoas muscle. A positioning needle is inserted anteriorly at the front 1/3 of the vertebral body, and the C-arm x-ray machine is used to adjust its position so that the anteroposterior view is at the level of the intervertebral space and the lateral view is at the front 1/3 of the vertebral body (Figure 2).

Subsequently, progressively expanding working casing was inserted until reaching the lateral side of the intervertebral disc at the lesion gap. A tubular dilator was then inserted to expand and serve as a working channel. After the removal of the dilators, a cold light source was connected. A sharp knife was used to incise the pushing gap, followed by alternating use of a hinge cutter, endplate scraper, nucleus pulposus forceps, and other tools to treat the intervertebral space. This process involved the removal of pus, granulation tissue, necrotic bone, residual intervertebral discs, sclerotic bone, etc., until normal bone tissue was reached. Samples were taken for bacterial culture, Next-Generation Sequencing (NGS), and pathological examination. The lesioned intervertebral space was repeatedly irrigated with copious amounts of hydrogen peroxide, povidone-iodine, and saline until the irrigation fluid was clear. The size of the intervertebral space was measured, a trial implant was placed, and the size of the fusion device was determined; a lateral interbody fusion device (Beijing Fule Technology Development Co., Ltd.) was vertically inserted through the working casing. The position of the fusion device and the restoration of intervertebral height were confirmed by C-arm x-ray fluoroscopy. After placing two drainage tubes (one as an irrigation tube and the other as a drainage tube), the working channel was removed in sequence. The incision was irrigated, hemostasis was thoroughly achieved, and the incision was sutured layer by layer.

#### 2.3.2 Posterior percutaneous pedicle screw fixation

The patient is positioned prone, and the C-arm x-ray machine is used to determine the position of the pedicles adjacent to the affected intervertebral disc. Lines are marked on the skin, followed by routine disinfection and draping. A small incision is made at the marked points, and a trocar is inserted. Under the guidance of a C-arm x-ray machine, the tip of the trocar is seen approaching the inner edge of the pedicle in the anteroposterior view, and in the lateral view, it enters the vertebral body. The trocar is then removed, and a guide wire is inserted. Pedicle screws are placed bilaterally along the guide wires. After the guide wires are removed, a connecting rod is installed. The position of the fixation is confirmed to be satisfactory under the C-arm fluoroscopy. The incision is irrigated, and after achieving thorough hemostasis, the incision is closed layer by layer.

### 2.4 Perioperative management

Upon admission, patients were empirically treated with moxifloxacin hydrochloride (Avelox) for anti-infection. Once surgical contraindications were ruled out, lesion puncture was performed under local infiltration anesthesia to obtain tissue samples for bacterial culture, next-generation sequencing (NGS), and pathological examination to identify the causative pathogen. Antibiotic therapy was adjusted based on the results of these tests and drug sensitivity assays. If a purulent infection was diagnosed, sensitive antibiotics were used; if Mycobacterium tuberculosis was identified, a combination therapy consisting of rifampicin, isoniazid, ethambutol, pyrazinamide, and Avelox was administered for anti-tuberculosis treatment. The duration of anti-infection therapy before surgery was 2 weeks.

During surgery, depending on the infectious pathogen, 0.5 g vancomycin or 0.3 g isoniazid is applied to the wound. Postoperatively, a continuous irrigation with either 3,000 ml of 0.9% sodium chloride injection plus 480,000 IU of gentamicin or 3,000 ml of 0.9% sodium chloride injection plus 0.6 g of isoniazid is administered daily for two weeks. The volume of wound irrigation drainage every 24 h is recorded. If leakage of the irrigation fluid is observed around the drainage tube, the irrigation is stopped and the drainage tube is promptly removed. Observation of clear drainage fluid along with significant reductions in white blood cell count, ESR, CRP, and procalcitonin levels may indicate the cessation of irrigation.

The use of postoperative antibiotics is determined by the results of bacterial cultures. If the cultures are positive, antibiotics to which the bacteria are sensitive are selected based on susceptibility test results. Intravenous administration of antibiotics is initially used, and then, once there is a significant decrease in CRP, ESR, WBC (every 3–5 days) and symptoms have significantly improved, treatment is switched to oral antibiotics to continue the anti-infection therapy, with a total course of at least 6 weeks. For tuberculosis patients, oral quadruple anti-tuberculosis medication treatment is continued postoperatively. After 3 months, pyrazinamide is discontinued, and the remaining three-drug anti-tuberculosis regimen is maintained for a total treatment duration of 12–18 months.

Patients are encouraged to wear a brace and engage in early ambulation post-surgery. Follow-up outpatient visits are scheduled at 1, 3, 6, and 12 months postoperatively to review thoracic spine x-rays (and thoracic spine CT or MRI if necessary), complete blood count, erythrocyte sedimentation rate, C-reactive protein, liver and kidney function tests, and other related examinations to evaluate the effectiveness of the anti-infection treatment and the status of bony fusion in the intervertebral space.

### 2.5 Efficacy evaluation

**Figure 3:**
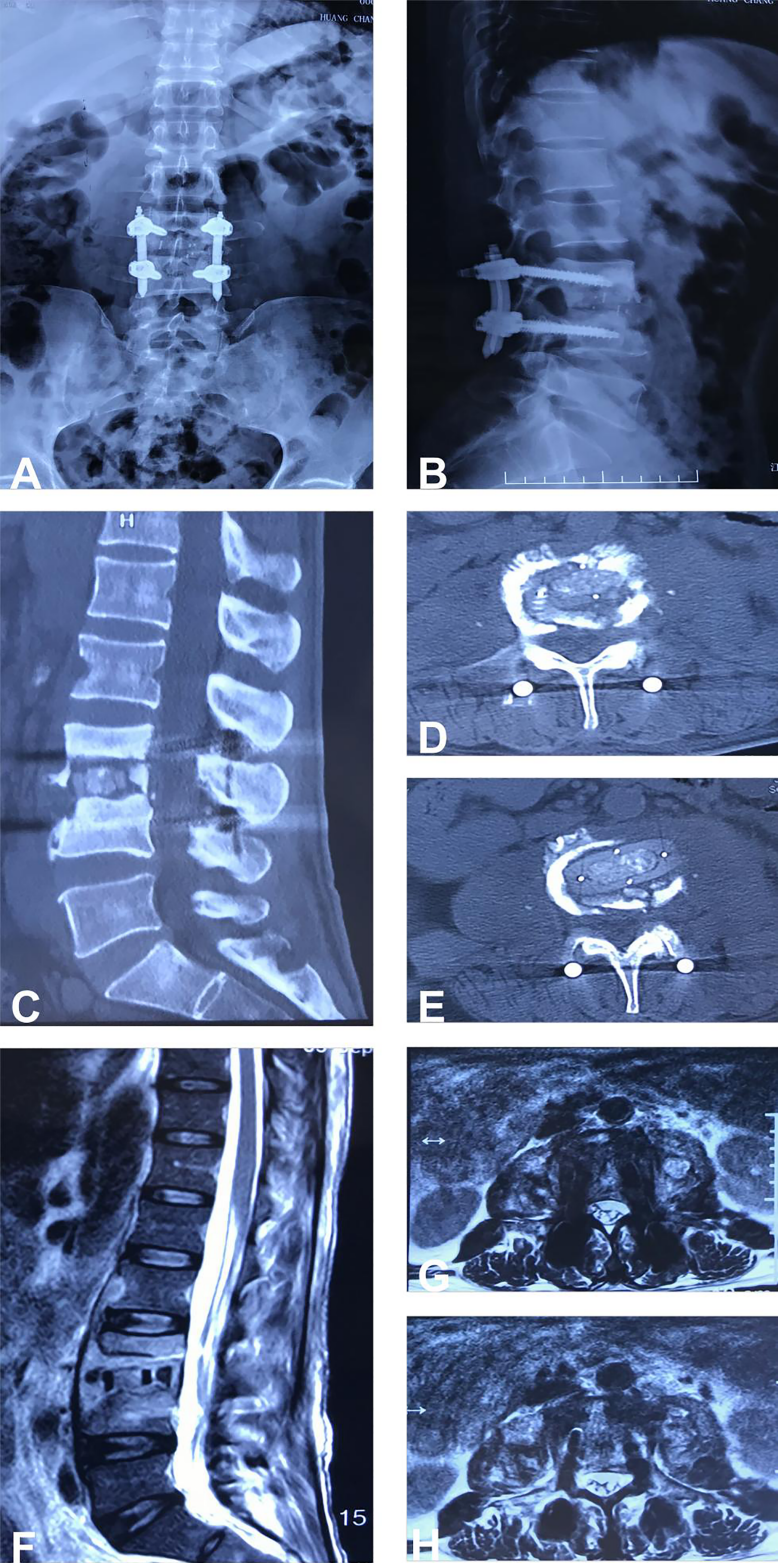
**(A–H)** Shows the postoperative follow-up x-ray, CT, and MRI results of the patient, demonstrating that the internal fixation is secure and effective, the position of the intervertebral fusion device is satisfactory, and the paravertebral abscess has been thoroughly eradicated.

The improvement of back pain was assessed using the Visual Analogue Scale (VAS) before and after surgery, while the Japanese Orthopaedic Association (JOA) score was used to evaluate the improvement of neurological function. White blood cell count (WBC), erythrocyte sedimentation rate (ESR), and C-reactive protein (CRP) were measured to assess the effectiveness of anti-infection treatment. Patients were followed up regularly after the surgery and lumbar spine x-rays and CT scans were reviewed to evaluate the status of internal fixation and bone graft fusion (Figure 3).

### 2.6 Statistical methods

The analysis was conducted using SPSS 22.0 statistical software. Quantitative data are expressed as mean ± standard deviation (x¯ ± s). Comparisons across different time points were made using repeated measures analysis of variance, with *P* < 0.05 indicating a statistically significant difference.

## 3 Result

**Figure 4:**
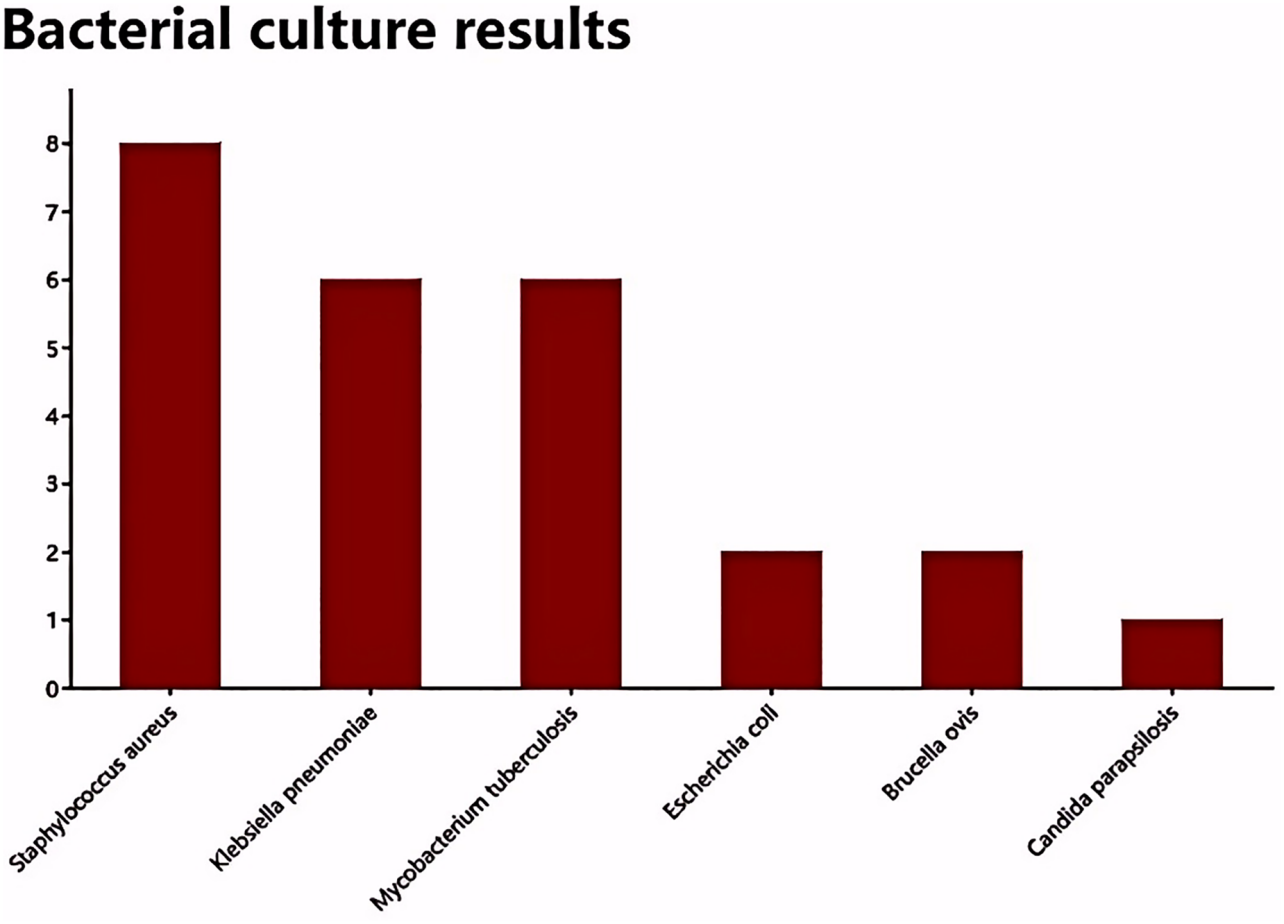
Presents the bacterial culture results for the infected tissues, which include 8 cases of Staphylococcus aureus infection, 6 cases of Klebsiella pneumoniae, 6 cases of Mycobacterium tuberculosis, 2 cases of Escherichia coli, 2 cases of Brucella, and 1 case of Candida glabrata.

All patients successfully completed the surgery, with operative times ranging from 180 to 310 min (average 254.7 ± 45.1 min) and intraoperative blood loss between 100 and 205 ml (average 163.3 ± 33.6 ml). There were no serious complications during or after the surgery for any of the patients. Postoperative symptoms of back pain and lower limb nerve damage showed improvement compared to preoperative conditions. Postoperative bacterial cultures of lesion tissues indicated infections with Staphylococcus aureus in 8 cases, Klebsiella pneumoniae in 6 cases, Mycobacterium tuberculosis in 6 cases, Escherichia coli in 2 cases, Acinetobacter baumannii in 2 cases, and Candida parapsilosis in 1 case (Figure 4). The postoperative pathological results were consistent with acute and chronic inflammatory responses as well as tuberculous granulomatous inflammation.

**Figure 5:**
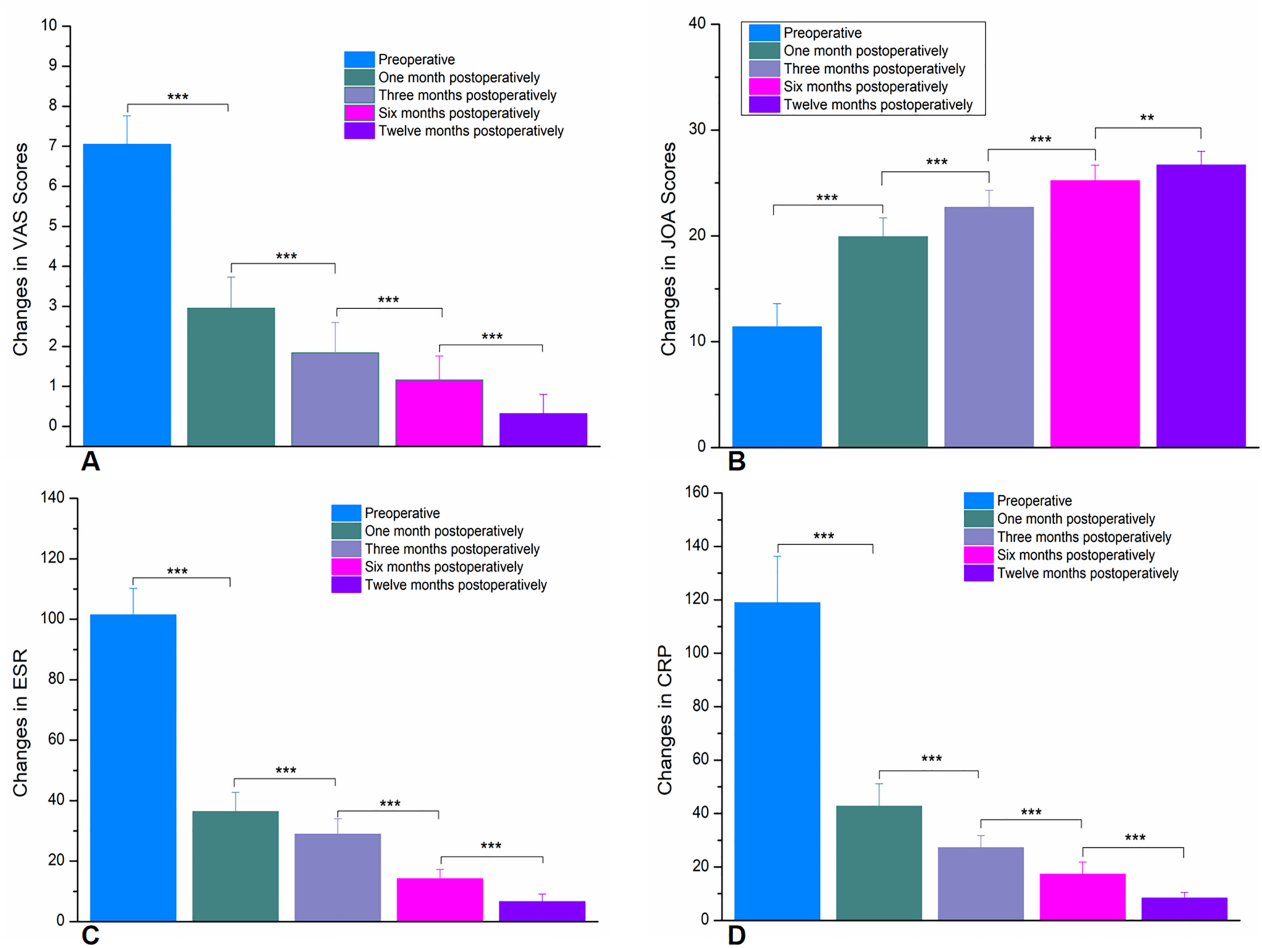
**(A)** Shows the changes in VAS scores preoperatively and at 1, 3, 6, and 12 months postoperatively. **(B)** Illustrates the changes in JOA scores preoperatively and at 1, 3, 6, and 12 months postoperatively. **(C)** Depicts the changes in ESR preoperatively and at 1, 3, 6, and 12 months postoperatively. **(D)** Presents the changes in VAS scores preoperatively and at 1, 3, 6, and 12 months postoperatively. (*** *p* < 0.0001,** *p* < 0.001).

All 26 patients were followed up for a period ranging from 12 to 24 months (average 18.3 ± 4.7 months). The postoperative scores for lumbar pain using the Visual Analog Scale (VAS), the Japanese Orthopaedic Association (JOA) scores, and the laboratory results for Erythrocyte Sedimentation Rate (ESR) and C-Reactive Protein (CRP) showed significant improvement at each time point (1, 3, 6, 12 months) compared to preoperative values (Figure 5), with the differences being statistically significant (*P* < 0.05). During the follow-up period, x-ray and CT scans were reviewed to observe whether the internal fixation was loose or broken. The success of bone grafting was assessed according to the criteria set by Moon et al. (6) for bone fusion. One patient experienced rod breakage at the 6-month postoperative follow-up, which was considered to be due to the patient's advanced age, severe osteoporosis, and collapse of the fusion device into the vertebral body, leading to an increased kyphotic angle and increased shear force on the rod, causing it to break. The remaining patients achieved bony fusion in their bone grafts 3–12 months (7.9 ± 3 months) after surgery.

## 4 Discussion

Despite the relatively low incidence of primary lumbar spondylodiscitis, the risk of infection has significantly increased due to the aging population, the rise in diabetes, and the increased use of immunosuppressants, leading to an uptick in the disease's incidence rate (1). Moreover, advancements in medical imaging technologies, such as MRI and CT scans, have allowed for the diagnosis of more asymptomatic or mildly symptomatic cases, which may also contribute to the reported increase in incidence rates in recent years (7). Primary lumbar spondylodiscitis can occur through multiple pathways, with hematogenous spread being the most common route of infection, typically caused by bacteria. Staphylococcus aureus, Klebsiella pneumoniae, and Mycobacterium tuberculosis are common pathogens (8). However, in recent years, due to the widespread use of antibiotics and the increase in bacterial resistance, the spectrum of infective pathogens is changing (7). For instance, the emergence of multidrug-resistant strains has made treatment more complex and challenging. Furthermore, non-bacterial pathogens, such as fungi and parasites, may also cause lumbar spondylodiscitis, especially in immunosuppressed patients. In this study, the most common pathogens were still Staphylococcus aureus, Klebsiella pneumoniae, and Mycobacterium tuberculosis, but non-bacterial pathogens, as described in the literature, were also found to cause lumbar spondylodiscitis.

Once a primary lumbar spondylodiscitis is diagnosed, it should be actively treated. In the early stages (with no or mild neurological deficits) and in the presence of severe comorbidities (limiting surgical options), conservative treatment is preferred (7). Studies have shown that the success rate of conservative treatment is about 90%, with the main methods including the use of sensitive antibiotics, pain management, bed rest, spinal brace fixation, and physical therapy (9). Although there are some treatment guidelines for primary lumbar intervertebral space infections, there is no unified standard. Instead, treatments are based on individualized principles. The main treatment goals are to eradicate the infection, alleviate pain, maintain spinal stability, and prevent further neurological dysfunction (9). Most patients with primary spondylodiscitis achieve satisfactory outcomes with non-surgical treatment, with reported clinical cure rates exceeding 80% (10). However, conservative treatment has a long duration, uncertain efficacy, and a risk of recurrence. Some patients do not improve after anti-infection treatment and may even experience progression of their condition, leading to deformities or symptoms of neurological damage (11, 12). Studies indicate that early surgical intervention may be more effective than conservative treatment, potentially reducing the risk of recurrence by 40% and the risk of death by 39%. Moreover, surgical intervention can more effectively clear the infection, promptly relieve spinal cord and nerve root compression, restore the normal spinal sequence, and rebuild spinal stability (11, 13).

There are multiple surgical treatment options available for primary lumbar spondylodiscitis, including: simple percutaneous pedicle screw fixation, simple drainage with tube irrigation, anterior focus debridement with bone graft fusion and internal fixation, lateral focus debridement with bone graft fusion and internal fixation, posterior focus debridement with bone graft fusion and internal fixation, combined anterior focus debridement with posterior internal fixation, and combined lateral focus debridement with posterior internal fixation (4). Although simple percutaneous pedicle screw fixation can stabilize the spine, the lumbar infection foci are not removed, and while simple irrigation drainage can control the lumbar infection foci, the lack of strong internal fixation may lead to vertebral collapse and spinal deformity (4). Anterior approaches may cause abdominal intestinal and urinary system damage, and if there are infection foci within the spinal canal, they cannot be removed (14). Moreover, posterior focus debridement can relieve dural sac compression and spinal canal stenosis, but there is a disadvantage that incomplete removal of the foci may lead to recurrent infection (4). Since Mayer introduced OLIF in 1997, it has become a mature minimally invasive technique in the field of spine surgery in recent years. This technique reaches the intervertebral disc through the space between the peritoneum and the psoas muscle, with the following advantages: Firstly, OLIF does not enter the spinal canal nor damage the posterior column of the spine, thus the risk of nerve root injury and venous plexus bleeding is lower; in addition, OLIF reaches the intervertebral disc through the anatomical space of the human body, which can reduce the damage to abdominal vessels and the urinary system; at the same time, OLIF does not require stripping of the psoas muscle, thereby avoiding damage to this muscle and the lumbar plexus nerves (5, 15–17). Research has found that OLIF combined with lateral or posterior pedicle screw fixation can reduce the subsidence rate. The subsidence rate of OLIF alone is reported in various literature to be approximately 15.6% to 36.3%, while the subsidence rate with combined pedicle screws is about 7.3% (18). Although the combined posterior pedicle screw procedure requires changing the surgical position and extends the operation time, there is no significant difference in hospital stay compared with lateral pedicle screw fixation (15). In addition, some scholars believe that OLIF combined with bilateral pedicle screw fixation might be the optimal solution, as it has stronger capabilities in maintaining lumbar stability, reducing graft subsidence, and maintaining intervertebral space height (5, 19).

In this study, we adopted the OLIF combined with posterior percutaneous bilateral pedicle screw fixation and lateral catheter irrigation and drainage. No serious complications occurred intraoperatively, and none of the patients experienced recurrent infection, subsidence of the interbody graft, or failure of the internal fixation during the follow-up period. All patients achieved bony fusion. Compared to other surgical techniques, the method used in this study has the following advantages: (1) This approach reaches the infected intervertebral space directly through the retroperitoneal space and psoas major muscle, resulting in smaller incisions and less trauma. Under the assistance of the access channel, the lesion area can be better exposed, and the debridement of the lesion is more thorough. (2) The debridement of the lesion in this technique does not require passing through the spinal canal, which can prevent the spread of infectious materials from the lesion into the spinal canal. (3) This technique allows for the insertion of larger interbody grafts, providing a greater bone graft contact area, which leads to a higher rate of postoperative bony fusion. (4) The use of lateral catheter irrigation in this technique enables more direct and comprehensive irrigation of the infected intervertebral space. This helps to further remove any residual purulent secretions, bacteria, inflammatory factors, and pain-causing factors from the intervertebral space during surgery, overcoming the limitations of systemic antibiotics in reaching the infected intervertebral space. (5) This study employs percutaneous posterior pedicle screw fixation, which involves smaller incisions and reduces the stripping of the psoas major muscle. At the same time, it avoids direct contact between the pedicle screws and the infected lesion. Compared to lateral screw fixation, this method allows for the extension or adjustment of the fixation segments according to the actual situation, resulting in a more robust and reliable fixation effect.

However, the surgical approach in this study still has some shortcomings. Firstly, the surgery requires a change of position intraoperatively, leading to a longer duration, increased intraoperative blood loss, and higher risk for patients with multiple underlying diseases and the elderly. Secondly, due to the prolonged surgery time, the duration of anesthesia and the use of anesthetic drugs are increased compared to other surgical methods, especially in elderly patients, which raises the probability of postoperative delirium.

In summary, the one-stage oblique lateral approach for debridement of lumbar spine infection foci, intervertebral bone grafting fusion, catheter irrigation and drainage combined with percutaneous pedicle screw fixation from the back is an effective surgical method for treating primary lumbar spondylodiscitis. It has the advantages of thorough debridement, ample bone grafting, safe operation, and reliable fixation, and can be considered a reliable treatment option for primary intervertebral space infections.

## Data Availability

The raw data supporting the conclusions of this article will be made available by the authors, without undue reservation.
